# Prevalence of Calcified Carotid Artery on Panoramic Radiographs in Postmenopausal Women

**DOI:** 10.5681/joddd.2009.011

**Published:** 2009-06-05

**Authors:** Jamileh Beigom Taheri, Mahkameh Moshfeghi

**Affiliations:** ^1^Associate Professor, Department of Oral Medicine, Faculty of Dentistry, Shahid Beheshti University of Medical Sciences, Tehran, Iran; ^2^Assistant Professor, Department of Oral and Maxillofacial Radiology, Faculty of Dentistry, Shahid Beheshti University of Medical Sciences, Tehran, Iran

**Keywords:** Calcification, carotid artery, panoramic radiography

## Abstract

**Background and aims:**

This study was designed to evaluate the prevalence of calcified carotid artery in 50 year-old and older postmenopausal dental outpatients for early diagnosis of individuals at risk of stroke.

**Materials and methods:**

This is a descriptive study of 200 panoramic radiographs. These radiographs included post-menopausal women referring to the Department of Oral Medicine at Shahid Beheshti Faculty of Dentistry during 2006-2007. The x-ray machine, developer and film type were the same for all the radiographs. Statistical analysis included chi-square test and Fisher’s exact test.

**Results:**

We found 22 calcified carotid arteries. The left and right carotid arteries were involved in 7 and 9 cases, respec-tively. In 6 cases both carotid arteries were calcified. Four individuals had no vascular risk factor excluding age and others had at least one risk factor. We found significant statistical correlation between hypertension, past history of myocardial infarction, and hypercholesterolemia with calcified carotid artery on panoramic radiographs.

**Conclusion:**

Under the limitations of the present study, prevalence of calcified carotid arteries is 11.0 % in 50 year-old and older postmenopausal dental outpatients.

## Introduction

Stroke or cerebrovascular accident has been reported to be the third leading reason for death in most countries after cardiovascular diseases and cancer. ^1^ Therefore, it is regarded an important public heath issue due to its high prevalence and high costs necessary for the physical and psychological rehabilitation of the patients. Reports have shown that approximately 60% of the patients who survive after a stroke suffer from a long-term physical and psychological disability. ^2^

It is obvious that stroke can be prevented; however, the major issue is to search for efficient methods of detection of stroke-prone patients. Strokes often occur as a result of atherosclerosis occurring in internal carotid artery. Dental panoramic radiography can detect this atherosclerosis. ^3^ Carotid artery calcification consists of a nodular mass or radiopaque vertical lines adjacent to or just below the intervertebral space at C3-C4 level on panoramic radiographs. ^4^

Friedlander and Lande ^4^ reported the possibility of identifying calcified atheroma plaques within the carotid artery on panoramic radiographs. Lewis and Brooks ^5^ suggested the use of panoramic radiographs to identify patients at risk of cerebral vascular accident (CVA). Cohen et al ^6^ warned that carotid artery calcification on panoramic radiographs is a major sign and marker for subsequent vascular risk and recommended that all patients with carotid artery calcification in their panoramic radiographs should be referred for vascular examination and treatment. Decrease in morbidity and mortality through early identification of stroke-prone patients has significant humanitarian and economic importance. ^3^

Panoramic radiography might prove to be significantly useful for the identification of patients at risk of stroke because signs of atherosclerotic disease may by visualized on panoramic radiographs. ^7, 8^ The most frequent finding is calcification at the bifurcation of the common carotid artery. ^7, 8^ Meanwhile, duplex ultrasonography is the most accurate technique for screening patients for carotid artery calcification for its non-invasive nature of the method but it is not cost-effective. ^9^ Dental practitioners must play their role in the issue and contribute to the early detection of patients at risk of CVA. Careful evaluation of dental panoramic radiographs by dental practitioners might be instrumental in saving patients’ lives.

Factors predisposing to carotid artery atherosclerosis include advancing age, male gender, systolic hypertension, hypercholesterolemia, cigarette smoking (more than 20 packs/year), diabetes mellitus, physical inactivity, obesity and coronary artery disease.^10^

In females, the incidence of myocardial infarction or other complexities of atherosclerosis is rarely reported except when accompanied by diseases like diabetes, hyperlipidemia or hypertension.^11^ Postmenopausal women are at higher risk of developing carotid artery atherosclerosis because they frequently develop an atherogenic blood lipid profile at the time menses ceases. Reduced levels of circulating estrogen are associated with an increase in hepatic lipase activity and a decrease in LDL catabolism, which result in increased levels of LDL and reduced levels of HDL.^12,13^ However, hormone replacement therapy can decrease the possible incidence of atherosclerosis and future mortality risk to some degrees.^11^

Current dental literature indicates that approximately 3-5% of patients exhibiting no systemic disease show carotid calcification on panoramic radiographs while the rate is 27-37% in patients with the history of atherosclerotic systemic diseases.^5,6,14,15^

Therefore, the present study was carried out to determine the incidence of carotid artery calcifications in postmenopausal women aged 50 and older referring to Shahid Beheshti University of Medical Sciences, Faculty of Dentistry, Department of Oral Medicine during 2007-2008.

## Materials and Methods

Two hundred individuals admitted to the Department of Oral Medicine, Faculty of Dentistry, Shahid Beheshti University of Medical Sciences during 2007-2008, were selected by census sampling technique and studied by observation and clinical examinations. The patients were postmenopausal women with at least 1 year after menses had stopped and were aged 50 and older. All the subjects needed panoramic views for their dental problems and none of them was exposed to panoramic radiography only for identifying carotid artery calcification in accordance with ethical considerations. The panoramic images studied had good diagnostic quality. Consent was obtained from all the patients studied.

The patients were questioned about cigarette smoking, the number of cigarettes smoked every day, the time menses had ceased, history of diabetes, hypertension, blood cholesterol level, and history of heart or brain strokes. Clinical examinations of fasting blood sugar (FBS) and fasting total serum cholesterol (FTSCH) within the past 6 months were used to validate the patients’ answers. In the case of a patient’s willingness, new tests of sugar and cholesterol were requested. The patients’ blood pressure was measured twice.

The patients using medications for blood cholesterol or with FTSCH > 200 mg/dL were considered hypercholesterolemiac. Also, those using antihypertensives and showing systolic blood pressure of 140 mmHg and more or diastolic blood pressure of 90 mmHg and more in two separate assessments were regarded hypertensive patients. Diabetic patients were also those using medicaments or those with FBS > 125 mg/dL.

The panoramic dental radiographs were obtained in the Department of Oral and Maxillofacial Radiology of Shahid Beheshti Faculty of Dentistry using Planmeca system (PM 2002 CC Proline, Finland) at 6-8 mA, 18 s, and the kilovolt peak range of 64 to 66 depending on an estimate of the subject’s jaw size according to standard protocol. Agfa Gavaret N.V Ortho CP-G Plus (15 × 30 cm) extraoral films and Kodak Lanex Regular intensifying screens were used. The films were taken by an automatic panoramic radiographic machine (Gendex Clarimat 300, USA) according to manufacturer’s instructions.

Radiographs with low diagnostic quality were excluded, i.e. no case of overexposure or underexposure was to be observed and cervical vertebrae of C3, C4 and C5 were to be observed. All radiographs were interpreted under the supervision of the study supervisor while the carotid artery calcification was considered as radiopaque nodular mass adjacent to the cervical vertebrae at or below intervertebral space C3-C4. No time limit was imposed and carotid artery calcifications were scored as present or absent. A questionnaire was completed for each patient and data was subjected to Fisher’s exact test for statistical analysis. All statistical analyses were performed by SPSS software Version 16 (SPSS Inc. Chicago, IL). Prevalence of carotid calcification was determined in samples and finally actual prevalence in society was assumed by a CI of 95%. Results were considered statistically significant at P < 0.05. The research committee of Shahid Beheshti Faculty of Dentistry reviewed and approved the present study protocol.

## Results

The subjects consisted of 200 postmenopausal women aged 50 years or older with the mean age of 60.65 years and standard deviation of 7.88 years (range: 50-84 years). Twenty-two patients (11.0%) showed carotid artery calcification while 178 patients (89.0%) did not demonstrate any calcifications. Nine calcifications (40.9%) were observed on the right side, 7 cases (31.8%) were detected on the left side and 6 cases (27.3%) were found on both sides.

Eighteen patients (9.0%) had a history of diabetes, 52 (26.0%) had hypertension, 5 (2.5%) were cigarette smokers with over 15 packs/year, 4 (2.0%) had suffered heart stroke and 36 (18.0%) demonstrated high blood cholesterol level. No case of CVA incidence was observed among the subjects.

Among 22 patients with carotid calcification, 18 cases showed at least one risk factor for cerebrovascular events while significant statistical differences were noted between incidence of carotid calcification and presence of at least one risk factor for these events (Table 1). Three subjects (16.7%) with diabetes involvement had carotid artery calcification while 19 patients (10.4%) without diabetes history showed calcification of carotid artery. No significant differences were found between the two groups, ruling out the role of diabetes involvement in the incidence of carotid calcification (P = 0.43).

**Table 1:** Incidence of carotid artery calcification according to the presence of at least one risk factor mentioned for cerebrovascular events

Carotid calcification risk factor	No calcification	Calcification	Total
No risk factor	108 (96.4%)	4 (3.6%)	112 (100%)
At least one risk factor	70 (79.5%)	18 (20.5%)	88 (100%)
Total	178 (89.0%)	22 (11.0%)	200 (100%)

P value = 0.004

Eleven subjects (21.2%) with a history of hypertension demonstrated carotid artery calcification while 11 cases (7.4%) without hypertension demonstrated carotid calcification with significant difference between the two groups (P < 0.01) (Table 2).

**Table 2:** Incidence of carotid artery calcification according to a history of hypertension in the subjects

Carotid calcification hypertension	Calcification	Total
Without hypertension	11 (7.4%)	148 (100%)
With hypertension	11 (21.2%)	52 (100%)
Total	22 (11.0%)	200 (100%)

P value = 0.01

Only 1 (20.0%) subject from 5 cases with smoking habits showed carotid calcification while 21 individuals having calcification did not smoke. Fisher’s exact test showed no significant differences between the two groups (P = 0.44).

Three individuals (75.0%) of those with a history of cerebrovascular events showed carotid calcification. However, 19 patients (9.7%) of those without any history of these events showed calcification. There was a significant relationship between carotid calcification and cerebrovascular accidents (P < 0.004) (Table 3).

**Table 3:** Incidence of carotid artery calcification according to a history of cerebrovascular events in subjects

Carotid calcification cerebrovascular events	No calcification	Calcification	Total
Without cerebrovascular events	177 (90.3%)	19 (9.7%)	196 (100%)
With cerebrovascular events	1 (25.0%)	3 (75.0%)	4 (100%)
Total	178 (89.0%)	22 (11.0%)	200 (100%)

P value = 0.004

As shown by Table 4, 9 individuals (25.0%) with a history of hypercholesterolemia showed carotid artery calcification while 13 individuals (7.9%) without hypercholesterolemia showed carotid calcification. Fisher’s exact test showed significant differences between the two groups regarding the incidence of carotid artery calcification (P < 0.007) (Table 4).

**Table 4:** Incidence of carotid artery calcification according to a history of Hypercholesterolemia in the subjects

Carotid calcification hypercholesterolemia	No calcification	Calcification	Total
Without hypercholesterolemia	151 (92.1%)	13 (7.9%)	164 (100%)
With hypercholesterolemia	27 (75.0%)	9 (25.0%)	36 (100%)
Total	178 (89.0%)	22 (11.0%)	200 (100%)

P value=0.007

Age distribution of the subjects was also studied. Of the patients aged 60 years old and lower, 14 individuals (12.5%) showed carotid artery calcifications on their panoramic radiographs while 8 individuals (9.1%) over 60 showed carotid calcification. No significant differences were found between the two age groups regarding the incidence of carotid artery calcification (P = 0.44).

## Discussion

Identification of carotid artery calcification on oral panoramic radiographs has been studied by researchers and dentists.^4^ The subjects have been 55 years old and older as they are more susceptible to carotid artery calcifications.

The present study assessed the incidence of carotid artery calcifications in postmenopausal women aged 50 and older referring to Shahid Beheshti Faculty of Dentistry during 2007-2008.

Cohen et al^6^ studied 1879 panoramic radiographs of males >55 years of age and reported 3.8% incidence rate for carotid artery calcification. Ahmad et al^16^ reported a calcification incidence rate of 11.4% in 500 panoramic radiographs. They showed similar incidence for both genders. Uthman and Al-Saffar ^17^ showed carotid calcification incidence rate of 38.8% in patients with stroke-related diseases and 11.6% in patients without stroke-related diseases with similar gender predilection. Tanaka et al^18^ evaluated 659 panoramic radiographs from 262 male and 397 female 80-year-old subjects and reported 4% carotid artery calcification incidence. The related incidence among females was 6.2%. Mehran^19^ showed 1.4% incidence rate of calcification in 700 patients. Friedlander and Altman^7^ assessed panoramic radiographs and medical records of 52 females and reported a carotid calcification prevalence of 31%. The results of the present study revealed an 11.0% incidence rate of carotid artery calcification in 200 postmenopausal women. Differences in the incidence of carotid artery calcification rates in different studies may be related to differences in gender, race and the time devoted to them. It must be noted that carotid artery calcification must be located in radiography field in order to be viewed on the radiograph. Ignoring this fact may result in incorrect assignment of patients as having or not having calcification.

The present study showed an incidence rate of 40.9% of carotid artery calcification on the right side, 31.8% on the left side and 27.3% on both sides while no significant differences were observed between calcifications occurring on both sides (P = 0.72). A non-significant statistical difference was seen between the two sides in a study carried out by Uthman and Al-Saffar ^17^ on men and women while they reported a higher incidence of bilateral calcification compared to unilateral calcification (40 cases vs. 21 cases). This finding does not coincide with the results of a study carried out by Ohba et al^20^ who reported calcification involvement in a selected Japanese sample to be 3 times more prevalent on the right side than on the left side (74% vs. 26%). Mehran^19^ reported 5 cases on the left side, 3 cases on the right, and 2 cases on both sides from a total of 10 carotid calcifications identified. The observed differences may be related to race, gender, and sample size of the populations studied.

Cohen et al^6^ found that 86% of the identified carotid artery calcifications are observed in patients with at least one risk factor for cerebrovascular disease with significant differences between the two groups. There were similar findings in the present study with 81% of patients with carotid calcifications exhibiting at least one related factor in their past medical history.

Cigarette smoking functions as a risk factor for cerebrovascular infarctions through three mechanisms: atheromatous atherosclerosis of the arterial wall,^21^ platelets agglutination in small vessels,^22^ and cardiogenic thrombosis due to arrhythmia or cardiac dysfunction such as myocardial infarctions. ^23^

Kumagai et al^24^ showed a significantly higher incidence of carotid artery calcification in male smokers than non-smokers on panoramic radiographs; however, similar relationship was not seen in females. Ahmad et al^16^ did not report this finding, either. No significant relationship was found between smoking and incidence of carotid artery calcification in the present study (P = 0.44). In order to clarify the full influence of smoking on carotid arteries, large sample size in needed as only 5 patients used to smoke in our study.

Significant differences were found between the incidence of carotid artery calcifications and history of hypertension in our study (P < 0.01). Friedlander and Altman^7^ showed that hypertension is a major risk factor for carotid artery atheromas in postmenopausal women, reporting significant relationships between hypertension and carotid artery calcifications. Uthman and Al-Saffar^17^ showed similar relationships in a sample of Iraqi patients without significant differences between males and females. However, Tanaka et al^18^ did not report any relationship between the presence of carotid artery calcification on panoramic radiographs and diastolic or systolic blood pressure, which might be attributed to race and age of patients under study.

In the present study, significant differences were observed between high blood cholesterol level and presence of carotid calcification (P < 0.007). This finding was expected since the main mechanism of atherosclerosis is an increase in the intima thickness and fat accumulation of mainly cholesterol and its esters. Uthman and Al-Saffar^17^ categorized patients with fasting total serum cholesterol > 200 mg/dL as hyperlipidemia cases, showing significant relationship between calcifications and hyperlipidemia (P < 0.01). This relationship was reported by Tanaka et al ^18^ to be due to small sample size (P = 0.38). In addition, Friedlander & Altman^7^ did not report a significant relationship between high blood cholesterol level and presence of atheromas in carotid arteries in postmenopausal women, presumably due to limited sample size.

Diabetes did not result in a significant differences in the identification of carotid artery calcifications in the patients studied (P = 0.43). It must be pointed out that different laboratories had been referred to for clinical tests by the patients with possible discrepancies in the results; patients had undergone only one blood sugar test (FBS) and time intervals existed between imaging and laboratory tests, all possibly having a role in the results obtained. Furthermore, FBS tests present only a snapshot of blood glucose at the time the test is performed and are unable to control glucose in the long run as glycated Hb assays do during a 6-8 week period. Larger, multi-center studies with accurate tests of blood sugar control and similar laboratory circumstances are needed to fully assess the relationship between diabetes history and carotid artery calcifications visualized on panoramic radiographs.

Different studies have been unable to demonstrate significant differences between a history of diabetes mellitus and carotid artery calcification separately. ^7,16,18^ However, Uthman and Al-Saffar^17^ assigned diabetic and hyperlipidemia patients to a metabolic group showing significant differences regarding the increased incidence of carotid artery calcifications with the presence of metabolic diseases. Friedlander and Maeder^25^ compared 49 diabetic patients aged 55 years old and higher with no history of myocardial infarction (MI) and CVA with 314 controls discovering carotid artery calcifications in 20.4% of diabetic and 0.6% of non-diabetic patients. The two groups were matched in this study regarding age and blood pressure and diabetic patients showed higher LDL, triglyceride and FBS while different values of total blood cholesterol were noted in the two groups.

From the 4 women with the MI history, 3 cases showed carotid artery calcifications on panoramic radiographs, demonstrating a statistically significant relationship between the presence of carotid artery calcification and MI factor. As carotid artery atheromas can lead to cerebrovascular and neurovascular events, this relationship was expected. Cohen et al^6^ argued that carotid artery calcification on panoramic radiographs should be considered an important marker for vascular risks as they showed higher end point vascular events (11% MI history and 7% CVA history) and 15% death rate in patients with carotid calcifications. However, Tanaka et al^18^ showed that the presence of carotid calcifications on panoramic radiographs might be related to the risk of subsequent vascular events of MI and CVA in a 5-year longitudinal study on Japanese 80-year-old patients. The results of this study were limited to the Japanese and a 5-year period while only less than 50% of the participants were enrolled for second examinations. Fukuto et al^26^ reported the incidence of carotid calcified atheromas to be 14.7 folds more in cases with a history of vascular diseases as compared with controls.

Some authors speculated that the advanced age of the patients might be a risk factor for visualization of carotid artery calcification on panoramic radiographs. ^10,16,19^ No similar relationships were found in the present study, possibly due to different age groups studied; the present study assessed 50-year-old patients and older while all aforementioned studies included individuals over 45. In addition, we studied only postmenopausal women in contrast to other studies. Studies with larger sample size might demonstrate significant differences between advanced age and presence of carotid artery calcifications.

The limitations of panoramic radiographs must be considered when identifying carotid artery calcifications as Madden et al^27^ showed panoramic radiography to be unreliable for detection of carotid artery calcifications compared to ultrasonography (sensitivity : 31.1%). Therefore, factors like obesity, smoking and hypertension might provide more information than carotid artery calcifications among dental patients. However, dental practitioners must be able to diagnose possible carotid artery calcifications and differentiate them from anatomic and pathologic forms as they have the opportunity to identify and refer for treatment patients at risk of vascular events.

## Conclusion

In the present study, carotid artery calcifications were detected in 11% of studied 50-year-old and older postmenopausal women referring to Shahid Beheshti Faculty of Dentistry during 2007-2008. A total of 80% of visualized carotid artery calcifications were seen in patients with at least one identified risk factor. Due to the presence of significant relationships between calcifications and some recognized risk factors it was speculated that detection of carotid artery calcifications on panoramic radiographs might be an important factor for identification and treatment of patients at risk of vascular events.

## References

[1] Fatahzadeh M, Glick M (2006). Stroke: epidemiology, classification, risk factors, complication, diagnosis, prevention and medical and dental management. Oral Surg Oral Radiol Endod.

[2] Friedlander AH (1995). Identification of stroke prone patients by panoramic and cervical spine radiography. Dentomaxillofac Radiol.

[3] Carter LC, Tsmidis K, Fabiano J (1998). Carotid calcifications on panoramic radiography identify an asymptomatic male patient at risk for stroke. Oral Surg Oral Med Oral Pathol Oral Radiol Endod.

[4] Friedlander AH, Lande A (1981). Panoramic radiographic identification of carotid arterial plaques. Oral Surg Oral Med Oral Pathol Oral Radiol Endod.

[5] Lewis DA, Brooks SL (1999). Carotid artery calcification in a general dental population: a retrospective study of panoramic radiographs. Gen Dent.

[6] Cohen SN, Friedlander AH, Jolly DA, Date L (2002). Carotid calcification on panoramic radiographs: an important marker for vascular risk. Oral Surg Oral Med Oral Pathol Oral Radiol Endod.

[7] Friedlander AH, Altman L (2001). Carotid artery atheromas in post-menopausal women: their prevalence on panoramic radiographs and their relationship to atherogenic risk factors. J Am Dent Assoc.

[8] Almog DM, Illig KA, Khin M, Green RM (2000). Unrecognized carotid artery stenosis discovered by calcifications on a panoramic radiograph. J Am Dent Assoc.

[9] Callow AD, Trachtenberg JD (1999). Diagnosis and surgical management of asymptomatic carotid stenosis. In: Ernst CB, Stanely JC, eds. Current Therapy in Vascular Surgery.

[10] Friedlander AH, Friedlander IK (1998). Identification of stroke prone patients by panoramic dental radiography. Aust Dent J.

[11] Fredrick JS, Ramzi SC. Blood vessels. In: Vinay KR: Robinsons Basic Pathology (Organ System). 7th Ed. 2003:14-7.

[12] Applebaum-bowden D, Mclean P, Steinmetz A (1989). Lipoprotein, apolipoprotein, and lipolytic enzyme changes following estrogen administration in postmenopausal women. J Lipid Res.

[13] Walsh BW, Schiff I, Rosner B, Greenberg L, Ravnikar V, Sacks FM (1991). Effects of postmenopausal estrogen replacement on the concentrations and metabolism of plasma lipoproteins. N Engl J Med.

[14] American Dental Association Council on Scientific Affairs. The use of dental radiographs: update and recommendations. J Am Dent Assoc 2006;137:1304-12. 10.14219/jada.archive.2006.0393 16946440

[15] Almog DM, Horev T, Illig KA, Green RM, Carter LC (2002). Correlating carotid artery stenosis detected by panoramic radiology with clinically relevant carotid artery stenosis determined by duplex ultrasound. Oral Surg Oral Med Oral Pathol Oral Radiol Endod.

[16] Ahmad M, Madden R, El-Ashiry K (2002). Prevalence of carotid calcified atherosclerotic plaques on panoramic radiographs. J Dent Res.

[17] Uthman AT, Al-saffar AB (2008). Prevalence in digital panoramic radiographs of carotid area calcification among iraqi individuals with stroke- related disease. Oral Surg Oral Med Oral Pathol Oral Radiol Endod.

[18] Tanaka T, Morimoto Y, Ansai T, Okabe S, Yamada K, Taguchi A, Awano S (2006). Can the presence of carotid artery calcification on panoramic radiographs predict the risk of vascular diseases among 80-year-olds. Oral Surg Oral Med Oral Pathol Oral Radiol Endod.

[19] Mehran M. Evaluation of carotid artery calcification on panoramic radiographs of patients over 45 years referral to Oral and Maxillofacial Radiology department of Dentistry of Shahid Beheshti University, 2002. Tehran: Shahid Beheshti University of Medical Sciences [Thesis].

[20] Ohba T,  takatay  TAKATAY, Ansai T, Morimoto Y, Tanaka T, Kioto S (2003). Evaluation of carotid artery atheromas detected by panoramic radiograph among 80 year olds. Oral Surg Oral Med Oral Pathol Oral Radiol Endod.

[21] Prati P, Vanuzzo D, Casaroli M, Bader G, Mos L, Pilotto L, Canciani L, Ruscio M, Touboul PJ (2006). Determinants of carotid plaque occurrence. a long-term prospective population study: the san daniele project. Cerebrovasc Dis.

[22] Yasue H, Hirai N, Mizuno Y, Harada E, Itoh T, Yoshimura M, Kugiyama K, Ogawa H (2006). Low-grade inflammation, thrombogenicity, and atherogenic lipid profile in cigarette smokers. Circ J.

[23] Cirillo P, De ROSA S, Pacileo M, Gagiulo A, Leonardi A, Angri V, Formisano S, Chiariello M (2006). Nicotine induces tissue factor expression in cultured endothelial and smooth muscle cells. J Thromb Haemost.

[24] Kumagai M, Yamagishi T, Fukui N, Chiba M (2007). Long-term cigarette smoking increases the prevalence of carotid artery calcification seen on panoramic dental radiographs in male patients. Tohoko J Exp Med.

[25] Friedlander AH, Maeder LA (2000). The prevalence of carotid artery atheromas on the panoramic radiographs of patients with type 2 diabetes mellitus. Oral Surg Oral Med Oral Pathol Oral Radiol Endod.

[26] Fukuto Y, Kimura T, Tatsuko M (2003). Oral findings of patients with cardiovascular disease: remaining tooth number and panoramic radiographic findings. Jpn J Oral Diag Oral Med.

[27] Madden RP, Hodges JS, Salmen CHW (2007). Utility of panoramic radiographs in detecting cervical calcified atheroma. Oral Surg Oral Med Oral Pathol Oral Radiol Endod.

